# Current Perspectives in Vaginal Laxity Measurement: A Scoping Review

**DOI:** 10.1055/a-2113-3202

**Published:** 2023-08-31

**Authors:** Indri Aulia, Michelle Valeria

**Affiliations:** 1 External Genitalia Section, Division of Plastic Reconstructive and Aesthetic Surgery, Department of Surgery, Dr. Cipto Mangunkusumo Hospital, Universitas Indonesia, Jakarta, Indonesia

**Keywords:** vaginal laxity, measurement, scoping review

## Abstract

This scoping review aimed to identify and categorize the available measurement options for vaginal laxity (VL), their indications of use, and whether these measurements can sufficiently provide objective clinical judgment for cases indicated for vaginal rejuvenation with many treatment options nowadays. Systematic searches were conducted on five electronic databases, manually searching articles' bibliographies and predetermined key journals with no date or study design limitations. We included all studies involving VL in their inclusion criteria, treatment indications, and outcome parameters. We used the Arksey and O'Malley frameworks as the guideline in writing this scoping review. Of the 9,464 articles identified, 66 articles and 11,258 subjects were included in the final analysis. The majority of studies were conducted in obstetrics and gynecology (73%), followed by plastic surgery (10%), medical rehabilitation (4.5%), dermatology (4.5%), and others (8%). Most studies originated from the North American region (30%). The following measurement tools were used: (1) interviews, (2) questionnaires, (3) physical/digital examinations, (4) perineometers, and (5) others. Our results suggested that subjective perception of laxity confirmed by directed interview or questionnaire is sufficient to confirm VL. Additional evaluation of pelvic floor muscle through digital examination or perineometer or other preferred tools and evaluation of sexual function through validated questionnaire (Female Sexual Function Index, Female Sexual Distress Scale-Revised, etc.) should follow to ensure holistic care to patients. Future research on the psychometric properties (reliability and validity) of commonly used measurements and the correlation in between subjective and objective measurements should be initiated before their clinical applications.

## Introduction

Vaginal laxity (VL), or loosening of the vagina, is often underreported by almost 80% of women. 1 2 The presence of VL complaints may be accompanied by significant problems in women's sexuality and further disturb their sense of well-being. 2 3 Qureshi et al reported a prevalence of VL as 1 in 6 women attending a plastic surgery center. 4 Similarly, Dietz et al reported a prevalence of 24% of patients reporting VL in their urogenital clinic. 5 Even if it is common and raises some concerns, many societies still consider women with complaints of loosening of the vagina as taboo. 3

Surgical and nonsurgical aesthetic treatments for VL are gaining more popularity lately. 3 6 However, this rising trend is not accompanied with similar patient awareness of VL condition itself. Patients often come to consultation with no prior realization of their own state of laxity. It is common for patients to ask their doctor regarding the appropriate timing for vaginal rejuvenation, the exact indications, or whether it is necessary to be done. A previous survey among women aged 25 to 45 years old who had at least one vaginal delivery and changes in vaginal tone or sensitivity reported that these women did not know how to articulate their experience of VL, did not know the validation of their problem, concerned of being dismissed or misunderstood, yet 50% of which were interested in a nonsurgical vaginal tightening procedure. 7 Plastic surgeons suited to provide treatments for genital rejuvenation may expect to see more of these patients, thus laxity should be properly addressed based on scientifically proven measurements and validated instruments that justify symptoms and treatment indications. 8

Heterogeneous patient assessment tools are available in the market based on various theoretical bases and physician judgment. 8 Weighting the potential of growth yet heterogeneity in this field, we deemed it necessary to identify and scope the available measurement options for VL. Therefore, we decided to conduct a scoping review that differs from a systematic review. It explores rather than summarizes the evidence. A scoping review is considered a systematic approach to charting and mapping broad evidence into simplified categorizations. 9 This study undertakes a scoping review of research to identify and categorize available measurement options for VL, the indications, and whether these measurements can sufficiently provide objective clinical judgment for cases indicated for vaginal rejuvenation with many treatment options nowadays. Additionally, we aimed to establish their roles in vaginal rejuvenation.

## Methods

**Fig. 1:**
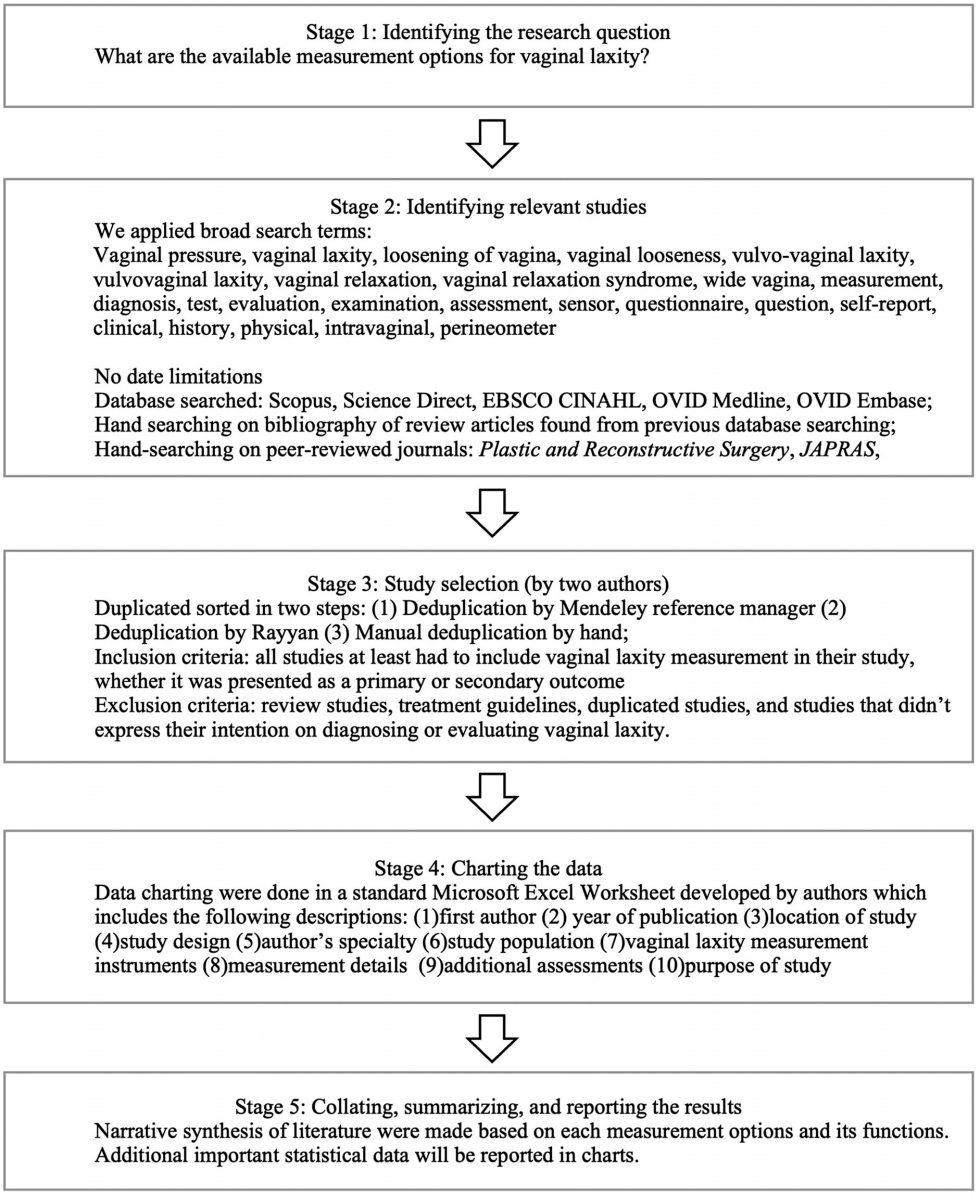
Framework for scoping review following The Arksey and O'Malley methodological framework. Methodological framework comprised of five stages, which are: (stage 1) identifying research question, (stage 2) identifying relevant studies, (stage 3) study selection, (stage 4) charting the data, (stage 5) collating, summarizing, and reporting the results.

We followed the Arksey and O'Malley framework for this scoping review. This framework was one of the first published guidelines for scoping reviews and has been widely used. The Arksey and O'Malley framework described five stages to conducting a scoping review with a goal primarily to identify gaps in the existing literature: (1) identifying the research question, (2) identifying relevant studies, (3) study selection, (4) charting the data, and (5) collecting, summarizing, and reporting the results ( Fig. 1 ). 9

**Fig. 2:**
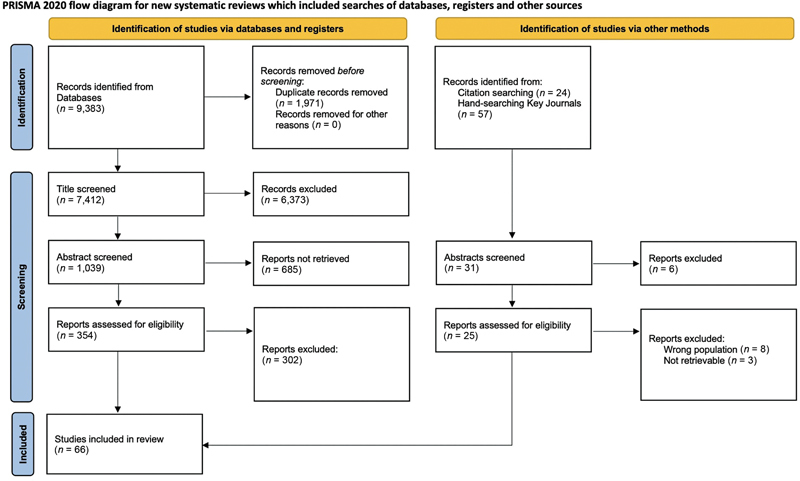
Flowchart of article identification, screening, selection, and inclusion. Article inclusion based on the Preferred Reporting Items for Systematic Review and Meta-analyses (PRISMA) 2020 guidelines.

The first stage involved establishing the research questions. Then, we directly conducted a systematic search of three different sources (five electronic databases, manually searching articles' bibliographies, and predetermined key journals). The identified articles were extracted and deduplicated using the Mendeley Reference Manager. Next, we used Rayyan, a Web-based tool that accommodates the simultaneous title and abstract screening process for the two authors. 10 All studies involved VL as their inclusion criteria, treatment indication, or outcome parameters. No limitations on the year of publication and study design were applied, as we aspired to find a broad level of evidence for this review. Exclusion criteria were as follows: review studies, treatment guidelines, duplicated studies, and studies that did not express their intention to diagnose or evaluate VL. Two authors independently reviewed the studies, and any disagreement was resolved through discussion. Complete searching strategies can be seen in Fig. 2 .

The final articles were then extracted into a predetermined worksheet in Microsoft Excel with the following details: study (author, year, design, country of origin), population (doctor-in-charge's specialty, subject of study), number of subjects, VL-related measurements, and other assessments. From which, we discovered the measurement options for VL: (1) interviews, (2) questionnaires, (3) physical/digital examinations, (4) photograph evaluations, (5) perineometers, and (6) others. Finally, a thorough quality analysis was conducted for each measurement.

## Results

### Study Selection and Characteristics

The primary search identified 9,464 articles, with 66 final articles and 11,258 subjects included in the final analysis ( Supplementary Table S1 , available in the online version). The articles were dated from 2009 to 2021 and written in English. In line with our inclusion criteria, all studies had to include VL measurement, whether it was presented as a primary or secondary outcome.

### Publication Demographics

The majority of studies were conducted in obstetrics and gynecology 48/66 (73%), followed by plastic surgery 7/66 (10%), medical rehabilitation 3/66 (4.5%), dermatology 3/66 (4.5%), and others 5/66 (8%). Twenty-five countries were represented across the following regions: North America, 20/66 (30%); Asia, 20/66 (30%); Europe, 13/66 (19%); South America, 7/66 (10%); Australia, 3/66 (4.5%); Africa, 1/66 (2%); and multiregions, 3/66 (4.5%).

### Questionnaires

A total of 45 studies with 8,391 subjects used questionnaires to assess VL ( Table 1 ). The International Consultation on Incontinence Questionnaire - Vaginal Symptoms (ICIQ-VS), electronic Personal Assessment Questionnaire for Pelvic Floor Disorders (ePAQ-PF), and Questionnaire for Diagnosing Open Vagina and Vaginal Flatulence (QUDOVVF) are validated questionnaires. Most of the studies used the Vaginal Laxity Questionnaire (VLQ 4 11 12 13 14 15 16 17 18 19 20 21 22 23 24 25 ; 16 studies), followed by other self-created questionnaires 26 27 28 29 30 31 32 33 34 35 36 37 (12 studies), ICIQ-VS 38 39 40 41 42 (5 studies), laser vaginal tightening 43 44 45 (3 studies), Visual Analog Scale (VAS 5 46 47 48 ; 4 studies), QUDOVVF 49 50 (2 studies), Patient Satisfaction Questionnaire 51 (1 study), Vaginal Functional Numeric Rating Scale (VFNRS 52 ; 1 study), and ePAQ-PF 1 (1 study).

**Table 1:** Vaginal laxity-related questionnaires

Questionnaires	Validated	Aims	Total subjects	Usage in study
VLQ	No	Perception on level of VL using 7-level of responses (Likert scale)	1,084	Treatment indication, treatment evaluation
ICIQ-VS	Yes	Presence and degree of bother of vaginal symptoms (including VL), sexual matters and impact on quality of life (QoL)	598	Treatment indication, treatment evaluation
LVT	No	Evaluation of vaginal tightness (patients' and partners'), treatment satisfaction, willingness to recommend treatment, and sexual gratification	376	Treatment evaluation
ePAQ-PF	Yes	Degree of bother on urinary, bowel, vaginal (including tightness), and sexual domains	2,621	Treatment indication
QUDOVVF	Yes	Diagnosis of VL and vaginal flatulence	232	VL diagnosis
VFNRS	No	Perception of degree of looseness in VL using a 4-level scale (0–3)	20	Treatment indication, treatment evaluation
PSQ	No	Perception of degree of looseness in VL using a 5-level scale and degree of sexual improvements posttreatment	364	Treatment indication, treatment evaluation
VAS	No	Subjective VL symptom bother from 0 (no bother at all) to 10 (worst conceivable bother).	1,910	VL diagnosis (degree of bother)

Abbreviations: ePAQ-PF, electronic Personal Assessment Questionnaire for Pelvic Floor Disorders; ICIQ-VS, International Consultation on Incontinence Modular Questionnaire-Vaginal Symptoms; LVT, laser vaginal tightening; PSQ, Patient Satisfaction Questionnaire; QUDOVVF, Questionnaire for Diagnosing Open Vagina and Vaginal Flatulence; VAS, Visual Analog Scale; VFNRS, Vaginal Functional Numeric Rating Scale; VL, vaginal laxity; VLQ, Vaginal Laxity Questionnaire.

In addition to the questionnaire that explicitly evaluated VL ( Table 2 ), we also found other questionnaires that evaluated symptoms often associated with VL. Complaints regarding sexual function were evaluated using the Female Sexual Function Index (FSFI), Female Sexual Distress Scale-Revised (FSDS-R), Pelvic Organ Prolapse/Urinary Incontinence Sexual Questionnaire-12, Sexual Satisfaction Questionnaire, Stabbatsberg self-rating scale, McCoy Female Sexuality Questionnaire, and ICIQ - Female Sexual Matters Associated with Lower Urinary Tract Symptoms (ICIQ-FLUTSSex). Urinary incontinence was evaluated using the Incontinence Impact Questionnaire-7, ICIQ - Urinary Continence Short Form, ICIQ-FLUTSSex, and Questionnaire of Urinary Incontinence Diagnosis. The Pelvic Floor Disability Index-20 was used to evaluate bowel, bladder, and pelvic symptoms collectively.

**Table 2:** Other questionnaires

Questionnaires	Validated	Aims	Usage in study
FSFI	Yes	Assess the sexual functions (desire, arousal, lubrication, orgasm, satisfaction, and pain)	Treatment evaluation
FSDS-R	Yes	Evaluate the presence of female sexual dysfunction (FSD) based on the frequency of 13 conditions related to sexual relationship	Treatment evaluation
PSIQ-12	Yes	Assess the frequency of different sexual functions (desire, climax, excitement, satisfaction, pain, incontinence, bulging, negative emotional, partners' erectile problems, premature ejaculation, and comparison between current and past orgasm)	Treatment evaluation
PFDI-20	Yes	Assess presence and degree of bother of certain bowel (CRAD-6), bladder/urinary distress inventory (UDI-6), or pelvic symptoms (POPDI-6)	Treatment evaluation
IIQ-7	Yes	Assess changes on activities, relationships, and feelings due to incontinence	Treatment evaluation
ICIQ-FLUTSSex	Yes	Assess changes on sexual function (pain due to dry vagina/during intercourse, urinary leakage during intercourse, degree of bother) associated with urinary incontinence	
ICIQ-UI-SF	Yes	Assess the frequency, severity, and impact on quality of life due to urinary incontinence	
SSQ	No	Assess subjects' level of satisfaction from vaginal intercourse	Treatment evaluation
Stabbatsberg self-rating scale (translated)	No a	Assess the current and comparison past-now sexual interest, sexual activity, sexual life, and pleasure during sex (total 8 questions, each question rated based on 5-level scale)	Treatment evaluation
MFSQ	Yes	Assess the female sexuality (frequency, enjoyment, satisfaction, arousal during sexual activity and orgasm, frequency of sexual thoughts, level of sexual interest, dryness/need of lubricant, satisfaction, pain, partner's erectile problems)	Treatment evaluation
QUID	Yes	Assess frequencies of stress and/or urge of UI symptoms	Treatment evaluation

Abbreviations: CRAD-6, Colorectal-Anal Distress Inventory 6; FSDS-R, Female Sexual Distress Scale-Revised; FSFI, Female Sexual Function Index; ICIQ-UI-SF, International Consultation on Incontinence Questionnaire-Urinary Continence Short Form; IIQ-7, Incontinence Impact Questionnaire-7; ICIQ-FLUTSSex, International Consultation on Incontinence Questionnaire-Female Sexual Matters Associated with Lower Urinary Tract Symptoms; MFSQ, McCoy Female Sexuality Questionnaire; PFDI-20, Pelvic Floor Disability Index (PFDI-20); POPDI-6, Pelvic Organ Prolapse Distress Inventory 6; PSIQ-12, Pelvic Organ Prolapse/ Urinary Incontinence Sexual Questionnaire; QUID, Questionnaire of Urinary Incontinence Diagnosis; SSQ, Sexual Satisfaction Questionnaire; UDI-6, Urinary Distress Inventory 6; UI, urinary incontinence.

a Translated from Stabbatsberg self-rating scale (validated).

### Medical Interviews

Medical interviews were chosen as a method to diagnose or evaluate VL in 19 studies, which involved 4,409 subjects ( Table 3 ). 5 45 46 47 48 53 54 55 56 57 58 59 60 61 62 63 64 65 66 Eleven studies made a diagnosis effort of VL using interviews only. 46 47 53 54 55 57 58 59 60 61 63 Four studies chose interviews in combination with other measurement options, 48 56 62 65 and one study added the VL degree of bother evaluation. 5 Four studies reported treatment evaluation by conducting interviews. 45 64 65 66 Additionally, two qualitative studies by Millheiser et al and Kingsberg and Millheiser conducted focus group discussions (FGDs) to obtain subjects' perception of VL. 7 67

**Table 3:** Interviews that suggest VL

Study (year)	Wordings used in the interview	Description	Total subjects	Usage in study
Lauterbach et al (2021) 53	Sensation of VL accompanied with decrease in sexual sensation during intercourse	Self-reported primary complaints	81	Diagnosis
Alexander et al (2022) 54	Have you noticed VL or vaginal looseness?	VL as an accompanying symptom to POP	531	Diagnosis
Alexander et al (2020) 55	Not specified	VL as an accompanying symptom to levator avulsion	805	Diagnosis
Moore et al (2014) 56	Not specified	Self-reported primary complaints, causing vaginal dysfunction, and desiring vaginal rejuvenation	78	Diagnosis
Talab et al (2019) 58	Not specified	VL as an accompanying symptom	376	Diagnosis
Talab et al (2018) 57	Not specified	VL as an accompanying symptom to POP	135	
Elena et al (2020) 59	Not specified	Self-reported complaints	95	Diagnosis
Dietz et al (2018) 5	Have you noticed VL or looseness?	VL as an accompanying symptom to pelvic floor and lower urinary tract dysfunction	324	Diagnosis
Jamali et al (2014) 60	Not specified	VL as an accompanying symptom to candidates for elective colpoperineoplasty	76	Diagnosis
Al-Hamdani et al (2019) 61	Sensation of wide vagina	Self-reported primary complaints	20	Diagnosis
Mustafa et al (2020) 46	Have you noticed vaginal laxity or looseness?	VL as an accompanying symptom to pelvic floor symptoms and lower urinary tract dysfunction	1,050	Diagnosis
Gaviria et al (2017) 48	Not specified	Self-reported primary complaints	45	Diagnosis
Cheng et al (2021) 62	Not specified	Self-reported complaints	47	Diagnosis
Ahmed et al (2019) 63	Desire to increase vaginal tightness and decreased sexual sensation a	Self-reported complaints	30	Diagnosis
Manzini et al (2020) 47	Have you noticed VL or vaginal looseness?	VL as an accompanying symptom to pelvic floor symptoms and lower urinary tract dysfunction	490	Diagnosis
Ostrzenski (2014) 64	Sensation of wide vagina at the vaginal opening	Self-reported primary complaints	1	Diagnosis and treatment evaluation
Aguilar et al (2016) 65	Unpleasant feeling of a too wide vagina	Self-reported primary complaints, partners' report of not feeling vaginal walls during intercourse	1	Diagnosis and treatment evaluation
Ulubay et al (2016) 66	Sensation of a wide vagina (diagnosis)	Self-reported primary complaints	38	Diagnosis and treatment evaluation
Vizintin et al (2012) 45	Self- and partner-reported improvement of vaginal tightness sensation	Self-reported primary complaints	185	Treatment evaluation

Abbreviations: POP, pelvic organ prolapse; VL, vaginal laxity.

a It was not stated clearly in text whether the sexual sensation was considered as a part of VL diagnosis or accompanying condition.

### Physical Examination

Five studies included physical examination as a method of VL measurement. 29 62 65 68 69 Three studies used physical examination to evaluate symptom severity, 29 62 68 three studies used the examination for treatment evaluation by comparing the examination during pre- and posttreatment, 62 65 69 and one study used a digital examination for VL diagnosis 69 ( Table 4 ).

**Table 4:** Physical examination

Study (year)	Examination	Total subjects	Usage in study
Aguilar et al (2016) ^65^	No explanation on method of examination (pretreatment) Physical examination showed a VL with preserved vaginal tonicity; pelvic muscle laxity with a diastasis of the anus elevators; no vaginal tissue defect (posttreatment) Physical examination showed a tighter vagina with a diminished vaginal caliber	1	Treatment evaluation
Cheng et al (2021) ^62^	(pre-treatment) Examination by using two fingers in which subjects were asked to squeeze these fingers to the highest degree possible in a lithotomy position • Light degree: strong pressure and could last more than 3 seconds • Moderate degree: less pressure lasting from 1-3 seconds. • Severe degree: nearly no pressure on 2 fingers and only a little pressure on 3 or more fingers (post-treatment) Confirmation of normal size of vaginal opening if two fingers can be inserted into the vagina	47	VL severity and treatment evaluation
Toplu et al (2021) ^29^	(pre-treatment) Examination using fingers to determine degree of VL • Slightly loose: at least 2 fingers were required to feel the vaginal tightness during bimanual examination • Moderately loose: at least 3 fingers were required to feel the vaginal tightness during bimanual examination • Very loose: 4 or more fingers were required to feel the vaginal tightness during bimanual examination	30	VL severity
Lee (2014) ^68^	(pretreatment) Examination using fingers to determine degree of VL into good, moderate, poor, and very poor (no further examination on definition of each severity)	30	VL severity
Abedi et al (2013) ^69^	(pr-treatment) Examination by using two fingers in which subjects were asked to squeeze these fingers to the highest degree possible in a lithotomy position. When the pressure tone could not be maintained for 3 seconds, VL was confirmed (posttreatment) Confirmation of normal size of vaginal opening with two fingers able to be inserted into the vagina	86	VL diagnosis and treatment evaluation

### Photograph Evaluation

Vaginal anatomical evaluation using photographs was described in three studies. 50 51 70 A standardized photograph procedure was chosen by the authors usually to define reliable and comparable visual information.

Mitsuyuki et al applied standardized photograph guidance to take before and after pictures. Then, an external independent evaluator determined which picture was the before/after for each patient and evaluated the degree of improvement using a Likert scale (0 = no change, 1 = mild, 2 = moderate, 3 = excellent change). 51 Mortiers et al stated that their method of objective evaluation using Photo Analysis for Diagnosing Open Vagina is reliable, reproducible, and valid for diagnosing open vagina or vaginal gaping. They took photographs of the vaginal opening (horizontal and vertical diameters and surface area) during rest, the Valsalva maneuver, and during pelvic floor contraction. 50 Meanwhile, the study by Watanabe et al was only obtained as a part of a conference abstract and mentioned before-after vaginal opening pictures as a mode of treatment effect evaluation, without further details on photography guides. 70

### Perineometer

A total of five studies with 294 subjects used a perineometer to evaluate the treatment effect on pelvic floor muscle (PFM) pressure ( Table 5 ). 28 40 45 63 68 A perineometer can be used to evaluate maximum and average PFM pressure, the maximum duration of the vaginal squeeze, and PFM endurance.

**Table 5:** Perineometer assessment

Study (year)	Brand	Description	Total subjects	Usage in text
Vizintin et al (2012) 45	Not mentioned	Improvement of maximal and average PFM pressure, duration of vaginal squeeze	17	Treatment evaluation
Lee (2014) 68	ExTT-101, APIMEDS Inc, South Korea	Improvement of maximal and average PFM pressure, duration of vaginal squeeze	30	Treatment evaluation
Sathaworawong et al (2022) 28	ExTT-101, Apimez Co., Ltd., Gyeonggi-do, Korea	Improvement of average PFM pressure	42	Treatment evaluation
Ahmed et al (2019) 63	Peritron (9300)	Improvement of PFM pressure	30	Treatment evaluation
Kolberg Tennfjord K (2016) 40	Camtech AS, Sandvika, Norway	Improvement of average of maximum PFM pressure and endurance	175	Treatment evaluation

Abbreviation: PFM, pelvic floor muscles.

### Other Measurements

Other evaluations reported in the included studies, such as vaginal tactile imaging (VTI), 71 vaginal biomechanic analyzer (VBA), 72 four-dimensional translabial ultrasonography (4D-TLUS), 5 32 46 54 55 surface electromyography (sEMG), 59 vaginal health index, 53 71 and other histological assessment, 68 are presented in Table 6 .

**Table 6:** Imaging and histological assessment

Measurements	Parameters (units)	Total subjects	Usage in study
VTI	Vaginal biomechanical parameter 1. Average vaginal tissue elasticity (mJ) 2. Vaginal tightening (kPA) 3. Contraction strength of pelvic muscles (kPA) 4. Reflex pelvic muscle contraction (kPA/mm)	25	Treatment evaluation
4D-TLUS	Measure of levator hiatal avulsion and overdistention	531	Treatment evaluation
VBA	1. Visualization and recording of vaginal wall deflection from camera attached to a vaginal probe 2. Quantification of maximum VL	13	VL diagnosis
sEMG	Measure of PFM electrical activity (µV) during PFM activations 1. Short contractions at maximum intensity 2. Sustained contraction and relaxation 3. Sustained contraction held as long as possible	95	Treatment evaluation
VHI	Histological scoring evaluating vaginal elasticity, fluid secretion type and consistency, pH, epithelial integrity, and moisture	106	Treatment evaluation
Histology	Improvement of mucosal architecture (thickness, number of cells) in epithelium and lamina propria	136	Treatment evaluation

Abbreviations: 4D-TLUS, four-dimensional translabial ultrasound; PFM, pelvic floor muscles; sEMG, surface electromyographic; VBA, vaginal biomechanic analyzer; VHI, vaginal health index; VL, vaginal laxity; VTI, vaginal tactile imaging.

## Discussion

This study is the first scoping review ever conducted on measuring VL and may represent a comprehensive coverage of literature discussing VL over the past decade (2009–2021).

### Subjective Measurement of Vaginal Laxity as a Symptom of Pelvic Floor Dysfunction

VL is a symptom of pelvic floor dysfunction that can be evaluated by subjective exploration, which varies from conducting interviews, utilizing validated questionnaires focusing on both VL symptoms and sexual function, to conducting digital examinations. Supported by a review article by the International Continence Society and International Urogynecological Association about terminologies for female pelvic floor dysfunction, VL was grouped under symptoms of sexual dysfunction, described as patients' complaint of excessive laxity of the vagina. 73 Forty-nine studies in this review also evaluated VL based solely on medical interviews and/or questionnaires. 73

Questionnaire is the most common assessment tool used for VL measurement. These questionnaires assess the presence of VL, either alone or in combination with one or more symptoms related to sexual, genitourinary, or gastrointestinal problems, in a scale-like form. Some validated questionnaires evaluating VL are the ICIQ-VS, 38 39 40 41 42 ePAQ-PF, 1 and QUDOVVF. 49 50 Despite the validated questionnaires mentioned above, a nonvalidated questionnaire, VLQ, has been used most often to report the symptoms and severity of VL. It has also been used to evaluate treatment indications and VL by different authors originating from multiple regions. The VLQ was first designed by Millheiser et al as a subjective tool to acquire subjects' perceptions of the level of VL/tightness using seven levels as responses (Likert's scale): very loose, moderately loose, slightly loose, neither loose nor tight, slightly tight, moderately tight, or very tight. The subjects' own perception of vaginal tightness/looseness was based on their own recall of status prior to vaginal deliveries. 19 Some other questionnaires that exclusively focus on laxity symptoms are VFNRS 52 and VAS. 5 46 47 48 These questionnaires help not only to confirm VL but also to measure the degree of severity, which is important for future treatment evaluation. 62 Based on our review, VLQ is sufficient to measure VL symptoms and the degree of bother subjectively.

### Vaginal Laxity and Its Relation to Pelvic Floor Muscle Function and Measurement

VL as a symptom may indicate disease or disruption to a normal anatomical or histological state that usually involves dysfunction in PFM. Signs accompanying the symptoms of VL should be explored when necessary. Trauma related to vaginal deliveries, especially with episiotomy and multiparity, is often correlated with damage to the levator ani muscle and causes the perception of increased VL and reduced PFM efficiency. 32 Meanwhile, hyperdistensibility or any disruptions to the levator ani muscle function may cause VL. 74 Khajehei et al reported vaginal looseness in 55% of women with a history of vaginal delivery with episiotomy. 27 This theory is the basis of PFM evaluation and treatment focusing on VL.

PFM function can be qualitatively evaluated by the tone at rest and strength during voluntary/reflex contraction. A validated grading system can categorize PFM strength into strong, normal, weak, or absent by digital palpation, electromyography, perineometer, or ultrasound. 73 Digital palpation based on compression created on the assessor's fingers was found in four studies in this review. 29 62 68 69 However, the method of examination and interpretation of the degree of symptom further varied between studies. In addition to digital palpation, other methods of PFM evaluation include objective measurements using external tools that quantify muscle pressure, electrical activities, or calculation based on images. Perineometers were used in all five studies for treatment evaluation to evaluate the before-after progression of PFM strength. 28 40 45 63 68 No studies set any cutoff pressure that may indicate as problematic and in need of intervention. Meanwhile, VTI and VBA are other tools to evaluate PFM strength and vaginal tightening based on pressure sensors. VTI can also combine and create a biomechanical mapping and visualization of the vagina and pelvis structures. 72 In VBA, a probe is attached to a camera to allow direct visualization of the vaginal wall. 71 sEMG has also been mentioned in literature as a method to evaluate PFM function; unfortunately, surface electrodes are nonselective due to the large surface area. 75 Another imaging device, 4D-TLUS, is used to record and assess the topography of the pelvis and vagina, therefore diagnosing levator avulsion and hyperdistension. 32 46 54 55

Photograph evaluation was used by Mortiers et al and Mitsuyuki et al in their studies to evaluate vaginal introitus through visual inspection. 50 51 Diagnosing VL using photographs, as described by Mortiers et al, 50 is reliable, reproducible, and valid. Photograph evaluation can also be combined with a Likert-like scale to observe the degree of improvement after treatment applications. 51 Another important finding from the study correlated validated questionnaire to validated photograph evaluation and showed that objective findings often do not correlate with subjective complaints; finding of VL through photographs was not accompanied by subjective complaints of VL, and women complaining of VL did not always have VL based on the photographs. 50

### Vaginal Laxity and Its Relation to Sexual Dysfunction

VL often becomes noticeable to women and/or partners during sexual intercourse, resulting in sexual dysfunction. Confirmed by the FGDs conducted by Kingsberg and Millheiser and Millheiser et al, apart from being subjectively perceived, VL causes significant distress, especially to subjects' emotions and sexual function. 7 67 Changes in VL are related to a decrease in vaginal diameter and frictional forces during intercourse, which is responsible for decreased sexual satisfaction. 4 45 Hence, it seems plausible to have sexual function evaluation prior to and after treatment. However, assessment of VL should be compulsory even before the sexual function evaluation. Presented in this review are some commonly used validated questionnaires, such as the FSFI, FSDS-R, and many others, as presented in Table 3 . Through measurement of both laxity and sexual function, suitable candidates for vaginal rejuvenation procedures and the treatment indications and targets would be made clear for both surgeons and patients, whether it is laxity that they want to repair, sexual function, or both.

In the future, routine vaginal examination prior to rejuvenation may include: (1) subjective validation of VL through directed interview or validated questionnaire (VLQ, VFNRS, etc.), (2) PFM evaluation through digital examination or perineometer or other preferred tools, and (3) sexual function evaluation through validated questionnaire (FSFI, FSDS-R, etc.). Further research on the psychometric properties (reliability and validity) of these measurements should be initiated before their clinical application in practice. In addition, studies focusing on finding the correlation between objective and subjective findings would create more justification for clinical practices. Though may be perceived similarly, VL is different with pelvic organ prolapse (POP) and genitourinary syndrome of menopause (GSM). The definition of POP clearly requires the presence of vaginal wall prolapse, which is not always present in VL. Additionally, a study by Alexander et al could not find any evidence of an early symptom of POP. 54 VL is also different from GSM in a sense that it is comprised of symptoms associated most commonly with postmenopausal hormonal changes in estrogen, including changes beyond laxity. 76
