# Supplementary material for: Current Perspectives in Vaginal Laxity Measurement: A Scoping Review
Source: Arch Plast Surg. 2023 Aug 31;50(5):452–62. doi: 10.1055/a-2113-3202 (PMC10556325; doi:10.1055/a-2113-3202)
Supplement: Supplementary file 1 — Supplementary Material [file 10-1055-a-2113-3202-s22oct0185rev.pdf]

Supplementary Table S1 Included studies

No	Study (year)	Type of study/level of evidence	Study population	No. of subjects	VL measurement	Measurement details	Additional assessment
1	Vicariotto et al (2017)[11]	Case series	Laxity, sexual dysfunction	25	VLQ	Diagnosis of VL based on VLQ treatment evaluation based on an increase from baseline of VLQ score	SSQ: sexual satisfaction evaluation PISQ-12: POP-related symptoms evaluation
2	Vicariotto (2016)[12]	Case series	Laxity, sexual dysfunction	12	VLQ	Diagnosis of VL based on VLQ questionnaire treatment evaluation based on mean changes in VLQ score	PISQ-12: POP-related symptoms evaluation SSQ: sexual satisfaction evaluation
3	Thomas (2018)[13]	Case series	Laxity, POP	20	VLQ	Diagnosis of VL based on VLQ Treatment evaluation based on mean changes in VLQ score	POP-Q: POP evaluation
4	Evans (2018) [14]	Case series	Laxity, sexual dysfunction	51	VLQ	Diagnosis of VL based on VLQ Treatment evaluation based on mean changes in VLQ score	FSDS-R: sexual satisfaction evaluation VAS: patient comfort evaluation
5	Molden (2018)[15]	case series	laxity, sexual dysfunction	46	VLQ	Diagnosis of VL based on VLQ score Treatment evaluation based on changes in VLQ score	SSQ: sexual satisfaction evaluation FSFI: sexual satisfaction evaluation GSQ: general satisfaction evaluation pelvic assessment: adverse event detection (infection) VAS: adverse event detection(pain)
6	Qureshi et al (2018)[4]	Prospective cohort	Laxity, sexual dysfunction	239	VLQ	Diagnosis of VL based on VLQ questionnaire	FSDS-R: sexual satisfaction evaluation FSFI: sexual function evaluation
7	Krychman and Tapscott (2018)[16]	RCT	Laxity, sexual dysfunction	155	VLQ	Diagnosis of VL based on VLQ score Treatment evaluation based on changes in VLQ score	FSFI: sexual function evaluation
8	Krychman et al (2017)[17]	RCT	Laxity, sexual dysfunction	186	VLQ	Diagnosis of VL based on VLQ Treatment evaluation based on changes in VLQ score	FSFI: sexual function evaluation FSDS-R: sexual distress evaluation
9	Thomas (2019)[18]	Case series	Laxity, UI, sexual dysfunction	30	VLQ and physical examination	Diagnosis of VL and treatment evaluation based on VLQ and physical examination (not explained)	FSFI: sexual function evaluation SSQ: sexual function evaluation UDI: UI evaluation IIQ: UI evaluation

 © 2023. The Author(s).

Supplementary Table S1 (*Continued*)

No	Study (year)	Type of study/level of evidence	Study population	No. of subjects	VL measurement	Measurement details	Additional assessment
10	Millheiser et al (2010)[19]	Case series	Laxity, sexual dysfunction	24	VLQ	Diagnosis of VL based on VLQ treatment Evaluation based on changes in VLQ score	SSQ: sexual satisfaction evaluation mv-FSFI: sexual function evaluation FSDS-R: sexual distress evaluation
11	Sekiguchi et al (2013)[20]	Case series	Laxity, sexual dysfunction	30	VLQ	Diagnosis of VL based on VLQ treatment Evaluation based on changes in VLQ score	FSFI: sexual function evaluation FSDS-R: sexual distress evaluation
12	Lalji and Lozanova (2017)[21]	Case series	Laxity, UI	27	VLQ	Diagnosis of VL based on VLQ treatment Evaluation of VL based on changes in VLQ score	ICIQ-UI SF : UI evaluation
13	Alinsod (2015)[22]	Case series	Laxity, UI	23	VLQ	Treatment evaluation based on mean changes in VLQ score	SSQ: sexual satisfaction evaluation
14	Wilkerson (2016)[23]	RCT	Laxity, sexual dysfunction	186	VLQ	Treatment evaluation based on mean changes from baseline in VLQ	FSFI: sexual function evaluation FSDS-R: sexual distress evaluation VALI: vaginal laxity-related sexual issues evaluation
15	Polland et al (2021) [24]	Prospective cohort	Laxity, POP	95	VLQ	Diagnosis of VL based on VLQ score	POP-Q: POP/GH evaluation FSFI: sexual satisfaction evaluation PFDI-20: POP measurement
16	Kim et al (2020)[25]	Case series	Laxity, sexual dysfunction	30	VLQ	Diagnosis of VL based on VLQ score Treatment evaluation based on changes in VLQ score	FSFI: sexual function evaluation
17	Almeida et al (2016) [26]	Cross-sectional	Laxity, sexual dysfunction	67	Self-created questionnaire	Diagnosis of VL based on questionnaire (adapted from ICIQ-VS)	Self-created questionnaire (adapted from FSFI): dyspareunia evaluation
18	Khajehei et al (2009)[27]	Case series	Laxity, sexual dysfunction	40	Self-created questionnaire	Diagnosis of VL based on questionnaire (VL-related symptoms)	Self-created questionnaire: sexual dysfunction evaluation
19	Sathaworawong et al (2022)[28]	Prospective cohort	Laxity, sexual dysfunction	42	Self-created questionnaire and perineometer	Treatment evaluation based on questionnaire (improvement of tightness sensation) Perineometer: PFM function evaluation	MFSQ: evaluate sexual activity and gratification evaluation

(*Continued*)

Supplementary Table S1 (*Continued*)

No	Study (year)	Type of study/level of evidence	Study population	No. of subjects	VL measurement	Measurement details	Additional assessment
20	Toplu et al (2021)[29]	Case series	Laxity, UI, sexual dysfunction	30	Self-created questionnaire and digital examination	Diagnosis and degree of severity of VL based on digital examination<br>Treatment evaluation based on questionnaire	QUID: UI evaluation<br>PISQ-12: sexual dysfunction evaluation
21	Jomah et al (2019)[30]	Case series	Laxity	39	Self-created questionnaire	Diagnosis of VL based on questionnaire (not specified)	Self-created questionnaire: degree of sexual satisfaction evaluation
22	Park et al (2015)[31]	Case series	Laxity, sexual dysfunction	187	Satisfaction scale (self-created question-naire)	Treatment evalua-tion based on 5-scale correction of VL	FSFI: sexual function evaluation
23	Thibault-Gag-non et al (2014)[32]	RCT	Laxity, POP, sexual dysfunction	294	Self-created questionnaire	Diagnosis of VL based on questionnaire	4D-TLUS to evaluate hiatal measurement and levator avulsion
24	Goodman et al (2010)[33]	Cohort retrospective	Laxity, sexual dysfunction	258	Self-created questionnaire	Diagnosis of VL based on FGPS questionnaire (feel-ing loose or "open") and doctors' perception	Questionnaire: all other sexual func-tions evaluation
25	Pardo et al (2006)[34]	Case series	Laxity, sexual dysfunction	53	Self-created questionnaire	Diagnosis of VL based on questionnaire	Self-created ques-tionnaire : loss of sexual satisfaction
26	Miklos (2014)[35]	Retrospective cohort	Laxity, sexual dysfunction, POP	130	Self-created questionnaire	Diagnosis of VL and treatment evalua-tion based on ques-tionnaire (modified from Pardo)	Self-created ques-tionnaire to evaluate POP and sexual function
27	Kim et al (2020)[36]	Cross-sectional	Laxity, sexual dysfunction	46	Self-created questionnaire	Diagnosis of VL from questionnaire (con-sisting 7-points scale identical to VLQ)	Self-created ques-tionnaire: dryness, pain, sexual satisfac-tion (patient and partner), arousal, and lubrication evaluation
28	Gaspar (2013)[37]	Case series	Laxity	N/A	Questionnaire (name not mentioned)	Treatment evalua-tion based on ques-tionnaire (improvement of VL symptoms)	Vaginal biopsy: adverse effect evaluation
29	Caruth (2018)[38]	Case series	Laxity, UI	30	ICIQ-VS	Treatment evalua-tion based on mean change from base-line of ICIQ-VS (frequency of VL symptoms)	IIQ-7 : evaluate the effect of accidental urine loss<br>ICIQ-UI-SF : urinary leakage and the degree of bother evaluation<br>PFIQ-7: pelvic floor condition
30	Madhu et al (2013)[39]	Case series	Laxity, QoL, sexual dysfunction	31	ICIQ-VS	Diagnosis of VL and treatment evalua-tion based on ICIQ-VS	ICIQ-VS: sexual func-tion, QoL evaluation

 © 2023. The Author(s).

Supplementary Table S1 (*Continued*)

No	Study (year)	Type of study/level of evidence	Study population	No. of subjects	VL measurement	Measurement details	Additional assessment
31	Kolberg Tennfjord et al (2016)[40]	RCT	Laxity, UI, sexual dysfunction	175	ICIQ-VS	Diagnosis of VL and treatment evaluation based on ICIQ-VS (frequency of VL symptoms)	ICIQ-FLUTSsex: sexual related symptoms evaluation USG and perineometer: pelvic muscle floor function evaluation
32	Lone et al (2015)[41]	Case series	Laxity, POP, UI	269	ICIQ-VS	Diagnosis and treatment evaluation based on mean changes seen in ICIQ-VS score	ICIQ-UI SF : UI symptoms evaluation POP-Q: POP staging evaluation
33	Ulrich et al (2015)[42]	Case series	Laxity, POP, UI	93	ICIQ-VS	Diagnosis and treatment evaluation based on mean changes in ICIQ-VS score	ICIQ-VS : vaginal symptoms and sexual life evaluation
34	Gaviria and Lanz (2012)[43]	Case series	Laxity, POP, UI, sexual dysfunction	21	LVT	Diagnosis based on interviews (complaint of VL) Treatment evaluation based on patients' and partners' LVT (improvement of tightness)	POP-Q: POP staging evaluation PISQ-12: POP, UI and sexual gratification evaluation
35	Gaviria et al (2016)[44]	Case series	Laxity, POP, UI, sexual dysfunction	103	LVT	Diagnosis based on interviews (complaint of VL) Treatment evaluation based on patients' and partners' LVT (improvement of tightness)	POP-Q: POP staging evaluation PISQ-12: POP, UI and sexual gratification evaluation
36	Vizintin et al (2012)[45]	Case series	Laxity, UI	252	Interviews, LVT, perineometer	Treatment evaluation based on interviews (self- and partner-assessment of vaginal tightness sensation), LVT questionnaires, and mean change in perineometer measurement (pelvic floor muscle strength)	MFSQ: evaluate sexual activity and gratification evaluation POP-Q: POP staging evaluation PISQ-12: sexual function evaluation FSFI: sexual function evaluation
37	Mustafa et al (2020)[46]	Case series	Laxity, POP, UI	1051	Interviews, VAS	Diagnosis of VL based on interview (statement of VL symptoms reported by patients), VL degree of bother based on VAS	ICS-POPQ : POP diagnosis 4D TLUS : pelvic floor dimension evaluation
38	Dietz et al (2018)[5]	Retrospective cohort	Laxity, POP, UI	324	Interviews and VAS	Diagnosis of VL based on interview (experience of VL), degree of bother based on VAS	ICS-POPQ : POP diagnosis 4D TLUS : pelvic floor dimension evaluation

(*Continued*)

 © 2023. The Author(s).

Supplementary Table S1 (*Continued*)

No	Study (year)	Type of study/level of evidence	Study population	No. of subjects	VL measurement	Measurement details	Additional assessment
39	Manzini et al (2020)[47]	Retrospective cohort	Laxity, UI	490	Interviews and scale (VAS)	Diagnosis of VL based on interviews (symptom of VL), degree of severity of VL based on VAS	POP-Q: POP staging evaluation<br>4D TLUS: hiatal measurement
40	Gaviria et al (2017)[48]	Case series	Laxity	45	VAS, perineometer	Diagnosis of VL based on VAS (0-10)<br>Perineometer: pre- and postvaginal pressure evaluation	VAS score: sexual gratification and lubrication evaluation
41	Neels (2019)[49]	Cross-sectional	Laxity, vaginal flatulence	209	QUDOVVF	Diagnosis of VL based on QUDOVVF	QUDOVVF : vaginal flatulence diagnosis
42	Mortiers et al (2018)[50]	Case series	Laxity	23	PHADOV, QUDOVVF	Diagnosis of VL using PHADOV and QUDOVVF	No additional assessment
43	Mitsuyuki et al (2020)[51]	Case series	Laxity	364	PSQ, photograph evaluation	Treatment evaluation based on changes in PSQ (question on VL) and before-after photograph	Assessment of sexual gratification using PSQ
44	Ostrzenski (2012)[52]	Case series	Laxity	20	VFNRS	Diagnosis of VL and degree of severity assessed based on VFNRS<br>Treatment evaluation based on changes on VFNRS	PISQ-12: sexual function evaluation<br>POP-Q: POP staging evaluation<br>USG: paravaginal defects confirmation<br>Modified body image scale: sexual and psychologic body image self-evaluation
45	Campbell et al (2018)[1]	Cohort prospective	Laxity, POP, UI, sexual dysfunction	2621	ePAQ-PF	Diagnosis of VL based on ePAQ-PF (questions on VL)	ePAQ-PF: POP, UI, sexual function evaluation
46	Lauterbach et al (2021)[53]	Case series	Laxity, sexual dysfunction	81	Interviews	Diagnosis of VL based on self-reported primary complaints	FSFI: sexual function evaluation<br>VHI: vaginal objective measurement
47	Alexander et al (2022)[54]	Case series	Laxity, POP	531	Interviews	Diagnosis of VL based on interview (symptom of VL)	Interview: vaginal lump or bulge and/or dragging sensation<br>POP-Q: POP diagnosis and staging<br>4D-TLUS: measure levator hiatal area (at rest and contraction)
48	Alexander et al (2020)[55]	Retrospective cohort	Laxity, levator avulsion	805	Interviews	Diagnosis of VL based on interview (symptom of VL)	ICS-POPQ: POP diagnosis<br>4D TLUS: levator avulsion diagnosis

Supplementary Table S1 (*Continued*)

No	Study (year)	Type of study/level of evidence	Study population	No. of subjects	VL measurement	Measurement details	Additional assessment
49	Moore et al (2014)[56]	Case series	Laxity, sexual dysfunction	78	Interviews and physical examination	Diagnosis of VL based on interviews (self-reported sensation of VL affecting sexual function) Confirmed with physical examination (not stated)	PISQ-12: sexual function evaluation
50	Talab et al (2018)[57]	Retrospective cohort	Laxity, POP, flatulence	384	Interviews	Diagnosis of VL based on interview (symptom of VL)	Interview: POP symptoms identification, flatulence diagnosis POP-Q: POP evaluation
51	Talab et al (2019) [58]	Retrospective cohort	Laxity, POP, flatulence	376	Interviews	Diagnosis of VL based on interview (symptom of VL)	POP-Q : POP evaluation interview : flatulence
52	Elena et al (2020)[59]	Prospective cohort	Laxity, UI, sexual dysfunction	95	Interviews	Diagnosis of VL based on interview (symptomatic patients who reported VL as a part of sexual dysfunction)	sEMG: PFM dysfunction detection PFIQ-7: UI, bowel, sexual dysfunction evaluation
53	Jamali et al (2014)[60]	Case series	Laxity, sexual dysfunction	76	Interviews	Diagnosis of VL based on interview (a complaint of vaginal laxity), treatment evaluation not mentioned	FSFI: sexual function evaluation
54	Al-Hamdani et al (2019)[61]	RCT	Laxity, sexual dysfunction	20	Interviews	Diagnosis of VL based on interview (statement of VL symptoms reported by patients)	Stabbatsberg sexual scale: sexual activity evaluation
55	Cheng et al (2021)[62]	Case series	Laxity, sexual dysfunction	47	Interview, digital examination	Diagnosis of VL based on interview (statement of VL symptoms), degree of severity of VL and treatment evaluation based on digital examination	FSFI: sexual function evaluation
56	Ahmed et al (2019)[63]	RCT	Laxity, sexual dysfunction	30	Interview, perineometer	Diagnosis of VL based on interview treatment evaluation based on changes in PFM measured with perineometer	SSQ: sexual satisfaction evaluation
57	Ostrzenski (2014)[64]	Case report	Laxity	1	Interviews	Diagnosis of VL based on interview	POP-Q: POP evaluation
58	Aguilar et al (2016)[65]	Case report	Laxity, sexual dysfunction	1	Interviews and clinical examination	Diagnosis and treatment evaluation of VL based on interview (unpleasant feeling of a too wide vagina) and clinical examination	Stabbatsberg scale: improvement in sexual life evaluation

(*Continued*)

 © 2023. The Author(s).

Supplementary Table S1 (*Continued*)

No	Study (year)	Type of study/level of evidence	Study population	No. of subjects	VL measurement	Measurement details	Additional assessment
59	Ulubay et al (2016)[66]	Cross-sectional	Laxity	38	Interviews	Diagnosis of VL by interview and treatment evaluation from interview (phone survey)	Phone survey : dyspareunia and partner's satisfaction evaluation (during phone survey/interview)
60	Kingsberg and Millheiser (2010)[7]	Cross-sectional	Laxity	50	Interviews and FGD	Diagnosis of VL based on interviews and FGD (prevalence of VL, related problems, patient's interest in undergoing vaginal rejuvenation)	No additional assessment
61	Millheiser et al (2010)[67]	Cross-sectional	Laxity, sexual dysfunction	50	Interviews and FGD	Diagnosis of VL based on interviews and FGD (prevalence of VL, patient's interest in undergoing vaginal rejuvenation)	Interview and FGDs: find the correlation of VL to sexual dysfunction
62	Lee et al (2014)[68]	Cohort prospective	Laxity	30	Digital examination, perineometer, interviews (partner), histology examination	Degree of VL status based on digital examination (mild, moderate, and severe), degree of VL and treatment evaluation perineometer to assess intravaginal pressure (maximum, average, and time), treatment evaluation based on partner's assessment of vaginal tightening, histology examination (hematoxylin and eosin stained specimens)	Postprocedural patients' assessment of sexual satisfaction and
63	Abedi et al (2014)[69]	Case series	Laxity	86	Digital examination	Diagnosis of VL made by digital examination (ability to squeeze and maintain pressure for 3 seconds) Treatment evaluation based on digital examination (direct postop examination with two fingers wide indicates tight vagina by operator)	FSFI: sexual function evaluation
64	Watanabe et al (2017)[70]	Case series	Laxity	50	Photograph evaluation	Treatment evaluation based on visual changes in photograph	No additional assessment

 © 2023. The Author(s).

Supplementary Table S1 (*Continued*)

No	Study (year)	Type of study/level of evidence	Study population	No. of subjects	VL measurement	Measurement details	Additional assessment
65	Lauterbach et al (2021)[71]	Prospective cohort	Laxity, sexual dysfunction	25	VTI	Treatment evaluation of VL based on VTI (vaginal elasticity and tightening)	FSFI: sexual function evaluation<br>VHI: objective vaginal health measurement
66	Zimmern et al (2021)[72]	Case series	Laxity	13	VBA	Diagnosis of VL from the measurement of vaginal wall deflection and visualization	No additional assessment

Abbreviations: 4D-TLUS, four-dimensional translabial ultrasonography; CRAD-6, Colorectal-Anal Distress Inventory 6; ePAQ-PF, electronic personal assessment questionnaire for pelvic floor disorders; FGD, focus group discussion; FSDS-R, Female Sexual Distress Scale-Revised; FSFI, Female Sexual Function Index; ICIQ-FLUTSSex, International Consultation on Incontinence Questionnaire – Female Sexual Matters Associated with Lower Urinary Tract Symptoms; ICIQ-VS, International Consultation on Incontinence Modular Questionnaire—vaginal symptoms; ICS-POPQ, International Continence Society Pelvic Organ Prolapse Quantification; ICIQ-UI-SF, International Consultation on Incontinence Questionnaire-Urinary Continence Short Form; IIQ-7, Incontinence Impact Questionnaire-7; LVT, Laser Vaginal Tightening; MFSQ, McCoy Female Sexuality Questionnaire; PFDI-20, Pelvic Floor Disability Index-20; PFM, pelvic floor muscle; PHADOV, Photo Analysis for Diagnosing Open Vagina; POPDI-6, Pelvic Organ Prolapse Distress Inventory 6; PSIQ-12, Pelvic Organ Prolapse/Urinary Incontinence Sexual Questionnaire-12; PSQ, Patient Satisfaction Questionnaire; QUDOVVF, Questionnaire for Diagnosing Open Vagina and Vaginal Flatulence; QUID, Questionnaire of Urinary Incontinence Diagnosis; RCT, randomized controlled trial; sEMG, surface electromyography; SSQ, Sexual Satisfaction Questionnaire; UDI-6, Urinary Distress Inventory 6; USG, ultrasound; VAS, Visual Analog Scale; VBA, vaginal biomechanic analyzer; VFNRS, Vaginal Functional Numeric Rating Scale; VHI, vaginal health index; VL, vaginal laxity; VLQ, vaginal laxity questionnaire; VTI, vaginal tactile imaging.

 © 2023. The Author(s).
